# Long-term outcome of chronic dialysis in children

**DOI:** 10.1007/s00467-007-0700-2

**Published:** 2009-03-01

**Authors:** Rukshana Shroff, Sarah Ledermann

**Affiliations:** Department of Nephrourology, Great Ormond Street Hospital for Children NHS Trust London, Great Ormond Street, London, WC1 N3JH UK

**Keywords:** Dialysis, Children, Long-term outcome, Vascular access, Cardiovascular mortality

## Abstract

As the prevalence of children on renal replacement therapy (RRT) increases world wide and such therapy comprises at least 2% of any national dialysis or transplant programme, it is essential that paediatric nephrologists are able to advise families on the possible outcome for their child on dialysis. Most children start dialysis with the expectation that successful renal transplantation is an achievable goal and will provide the best survival and quality of life. However, some will require long-term dialysis or may return intermittently to dialysis during the course of their chronic kidney disease (CKD). This article reviews the available outcome data for children on chronic dialysis as well as extrapolating data from the larger adult dialysis experience to inform our paediatric practice. The multiple factors that may influence outcome, and, particularly, those that can potentially be modified, are discussed.

## Mortality in children on dialysis

Survival data for paediatric patients on chronic dialysis from both national registries and single-centre studies can provide useful information. In this paper we have compiled data from large national registries, including the United States Renal Data System (USRDS), a compulsory registration system, which includes children ≤ 19 years; the North American Pediatric Renal Trials and Collaborative Studies (NAPRTCS), which allows voluntary data reporting and includes children ≤ 21 years; the United Network for Organ Sharing (UNOS), which collects data on all patients registered for renal transplantation in the USA; the European Renal Association–European Dialysis and Transplant Association (ERA–EDTA), a voluntary organization, which coordinates a national European registry for all patients; the Australia and New Zealand Dialysis and Transplant Association (ANZDATA), which is a comprehensive, compulsory database including children up to 20 years of age; and other national registries, including the United Kingdom Renal Registry, the National Dutch Registry, and the Italian Registry, and present the key points.

**Fig. 1:**
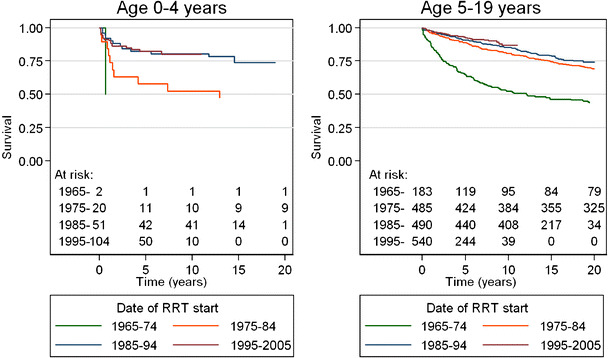
Kaplan–Meier graphs of overall rates of survival (stratified by age groups and decade during which renal replacement therapy began) among children and adolescents in Australia and New Zealand from 1965 to 2005. All patients were followed until death or the end of follow-up at 31 December 2005. The number of patients at risk at each time point is shown below each graph. We are grateful to Drs. Stephen McDonald and Jonathan Craig for providing this figure from the ANZDATA Registry

USRDS data for 2006 [1] report an adjusted mortality rate for dialysis patients (age 0–19 years) who started treatment between 1995 and 1999 of 56.5/1,000 patient years at risk. In their most recent report, NAPRTCS reviewed the survival data of 2,781 patients aged 0–21 years on long-term dialysis without a history of previous transplantation and whose index dialysis course was the first for the patient [2]. The patient survival rates were 95%, 90.1% and 85.7% at 1 year, 2 years, and 3 years, respectively (censored for transplantation or if lost to follow-up) [2]. Offering the longest longitudinal follow-up period over four decades, ANZDATA reports on the long-term survival rates of 1,634 children under 20 years of age who started renal replacement therapy (RRT) between 1963 and 2002 and can, therefore, uniquely offer 20-year survival data [3]. At 10 years, survival rate was 79%, and, at 20 years, it was 66% [3], Fig. 1.

Of the causes of death specified, cardiopulmonary disease was the reason cited most often, at 21.5% overall and for each specific age group [2]. The Dutch Registry data attributes 41% of deaths to cardiovascular disease and 21% to infection [4]. Similarly, ANZDATA reports that the most common cause of death of those on dialysis was cardiovascular disease (45%), with the second most common being infection (21%) [3]. Cardiovascular disease accounted for 57% of deaths in children on haemodialysis and 43% of those on peritoneal dialysis, compared with 30% in those with a functioning transplant in whom malignancy was responsible for 14% of deaths [3].

## Factors influencing outcome

### Overall mortality in dialysis patients compared with healthy children and those with transplants

Dialysis in children carries a significantly higher mortality rate than that for the age-adjusted population. In 2002 a Dutch Registry cohort study determined the mortality rate of 381 children with the onset of end-stage renal disease (ESRD) aged 0–14 years between 1972 and 1992 [4]. The overall mortality rate was 1.57/100 patient years for RRT patients, while the mortality rate standardized for age (which compares a specific age group in a certain year divided by the expected mortality in that age group in the same year) was 31 [4]. Similarly, in the larger ANZDATA report, mortality rates were 30-times higher than for children without chronic kidney disease (CKD) [3].

Dialysis treatment was also associated with a mortality risk more than four-times higher than for children who had received a transplant [3]. The UNOS data from 2002 report a mortality rate of 21/1,000 patient years for dialysis patients and 2/1,000 patient years for transplant recipients [5].

### Era of dialysis

ANZDATA reports a substantial improvement in the long-term survival rates of children and adolescents with CKD stage 5, with a mortality rate of 11 per 100 patient years between 1963 and 1972 and 1.8 per 100 patient years in those who started dialysis after 1993 [3]. The Dutch cohort reports a similar improvement, with survival rates of 81% and 79% at 5 years and 10 years for children on dialysis from 1972 to 1981 compared to 89% and 85%, respectively, in the 1982–1991 cohort, including those with a functioning graft [4].

However, the USRDS reports only a marginal improvement in survival during the 1990s, with 88.5% of patients who began dialysis between 1990 and 1995 surviving, compared to 89.2% survival for those who started dialysis from 1995–1999 [1]. The continued improvement in survival rates with time may be underestimated, as younger children and those with significant non-renal co-morbidity are taken onto dialysis programmes.

### Age at start of dialysis

All registries report a significantly higher mortality rate in infants starting dialysis. ANZDATA clearly identified younger age at the start as a risk factor, with a 5-year survival, if under 1 year at the start of RRT, of 73% compared to 86% for the whole population and a fourfold increased risk of death compared to children aged 15–19 years, Fig. 1 [3]. NAPRTCS report the highest mortality rate in children less than a year old at the start of dialysis, with survival rates of 83.2%, 74.3% and 66.4% at 1 year, 2 years and 3 years [2]. By further analysing the NAPRTCS database [6], we find that the outcome of dialysis initiated in the neonatal period (<1 month) is comparable to that of dialysis in children < 2 years age.

It must be kept in mind that treatment thresholds vary amongst different centres, reflecting the varying opinions amongst nephrologists towards offering RRT to the very young or those with co-morbidity [7]: the under 5-year-olds made up 22.9% of the overall cohort of paediatric dialysis patients in the NAPRTCS report [2] and 9.3% in the ANZDATA Registry [3]. In our study [8] of 98 children on long-term dialysis since 1984, 21 were < 1 year of age and 54 were < 5 years at initiation of dialysis. The overall rate of patient survival was 83%. The mortality rate was 2.7-times greater in children who required renal replacement therapy under the age of 5 years [8]. The condition of the anuric infant is particularly difficult to manage, and anuria has been discussed as an important risk factor in some studies [9].

Paediatric dialysis carries a significantly lower mortality rate than for older age groups on dialysis. The USRDS data for 2006 report a mortality rate for paediatric dialysis patients that is 25% that for adults on dialysis. Even young adults aged 20–44 years have a significantly higher mortality rate than children (ages 0–19 years), with 5-year and 10-year adjusted mortality rates of 68.1 vs 60.7 and 52.9 vs 31.6, respectively [1].

### Duration of dialysis

In adults the effect of long-term dialysis (vintage) on mortality risk is complex and influenced by co-morbidities and treatment factors. When adjusted for these factors, vintage is associated with increased mortality rate, although the rate has been reported to decrease after 8 years on dialysis, presumably due to early deaths of high-risk patients [1]. In contrast, the UK Renal Registry reports that the risk of death does not differ significantly with increasing length of time on dialysis except in those aged >65 years after 5–6 years on dialysis [10]. Although dialysis vintage is considerably shorter in children than in adults, cardiovascular morbidity has been clearly associated with the length of time on dialysis [11].

### Modality of dialysis

The Italian Registry data report on the survival of 458 children aged less than 15 years who started long-term dialysis [295 on continuous peritoneal dialysis (CPD)/163 on haemodialysis (HD)] with no preceding RRT from 1989–2000 [12]. Children less than 5 years old were almost exclusively managed with CPD, and such children had a poorer 5-year survival rate than children aged 5–15 years on either CPD or HD, for whom survival rates were not significantly different. This confirms the findings of Wong et al., in 2002, who used USRDS data and found no survival difference between dialysis modalities in children [13]. However, these data must be interpreted with caution, as, although some patients will elect HD as their primary choice of dialysis, it is often only offered when complications of peritoneal dialysis (PD) occur [2, 10], and, thus, children on HD may be older, have greater co-morbidity and a longer dialysis vintage than those on PD, making comparisons between the groups difficult.

### Co-morbidity

In recent years, patients with non-renal co-morbidity, such as multi-system involvement from inherited disorders, prematurity or CKD following overwhelming infection with multi-organ damage, increasingly have been offered renal replacement therapy. The importance of both renal co-morbidity, i.e. anuria, and non-renal co-morbidity, including pulmonary hypoplasia and severe developmental delay as significant risk factors for increased mortality in infants and young children, has been identified by analysis of NAPRTCS data [9] and in smaller studies [14–16]. The Italian multicentre study also reported a higher proportion of deaths in the presence of non-renal disease [12].

Our data on the long-term outcome of dialysis have shown that 30 children out of a cohort of 98 children on chronic dialysis had significant non-renal co-morbidity, including neuro-developmental delay, syndromes with multi-system involvement, congenital cardiac disease, malignancy and inherited metabolic disorders [8]. Of the 17 deaths in the study group, 76% were of those with associated co-morbidity, a 7.5-times greater risk of death in this group [8].

### Primary disease

The USRDS cites primary diagnosis as an independent determinant of mortality for children on dialysis, with those with glomerulonephritis and hereditary or congenital disease having a greater 5-year survival than those with secondary glomerulonephritis or vasculitis [1]. The poor transplant outcome associated with abnormal bladder function may be improved by prior augmentation cystoplasty, but there is, as yet, no data on the possible effects this may have on potential long-term PD [17].

As the age at initiation of dialysis and the presence of both renal and non-renal co-morbidity are unavoidable risk factors, it is essential that those caring for children on dialysis are aware of the potentially modifiable factors that might improve long-term survival and the quality of life for their patients. Although a successful transplant from a deceased donor or a live related donor is the goal for all patients with end-stage renal failure (ESRF) with confirmed survival benefits, episodes of dialysis or long-term dialysis may be necessary. It is, therefore, essential that dialysis access be preserved, peritoneal membrane function maintained, adequate dialysis delivered, metabolic complications prevented, and adequate nutrition and growth ensured. The cardiovascular risk factors must be minimized, and every support offered to ensure continuity of education for the best long-term outcome.

## Treatment factors that may influence outcome

### Dialysis access

The initial placement and subsequent conservation of either vascular or peritoneal access is critical for the young patient with CKD stage 5 in whom RRT may be a life-long undertaking. A NAPRTCS study in 2003 of 1,992 incident dialysis children showed that 70% had been started on PD and the remaining 30% on HD, with 96% of those < 2 years old having been started on PD. The NAPRTCS 2006 report shows that, although HD is being increasingly used as the incident dialysis modality, approximately 64% of children continued to start dialysis on PD [2]. In the NAPRTCS 2003 study, although 68% terminated dialysis as they had received transplants, 20% had changed dialysis modality over the 6-year study period. The majority of changes from HD to PD occurred within the first few months, but, in contrast, the change from PD to HD occurred more slowly and was mainly attributable to recurrent infections [18]. There was a high rate of PD catheter revision (45%), mainly due to catheter malfunction. The access in use after 6 months in children on HD was almost exclusively via percutaneous catheters in those aged less than 6 years, but 57% of those greater than 6 years of age were dialyzed using an arteriovenous (AV) fistula or graft. HD access revision rates were very high, with 919 revisions among the 584 initial placements, but 31% were for creation of more permanent access. Both the National Kidney Foundation–Dialysis Outcomes Quality Initiative (NKF-DOQI) and the UK Renal Association now have paediatric clinical practice guidelines and offer recommendations for optimal dialysis access and its preservation [19, 20].

#### Vascular access

Clinical practice guidelines clearly recommend that permanent access as either a native fistula or graft is preferred for most children on maintenance HD. If central venous catheters are used (i.e. in small or uncooperative children, where HD is initiated before a planned live related transplantation or in patients in whom early transplantation is anticipated), catheter size should be matched to patient size to minimize vessel trauma but allow sufficient flow for adequate HD. External cuffed access should be placed in the internal jugular vein, with the tip in the right atrium rather than the subclavian veins where the risk of stenosis is high. The right side is preferred, as there is decreased risk of thrombosis and the right side is usually contralateral to the non-dominant arm, which may eventually be needed for fistula formation. If possible, in all children with CKD stages 3–5, the use of the non-dominant arm for venipuncture and lines should be avoided [20, 21]. A study of adults has shown that HD with any type of venous catheter compared with a graft or fistula increases the risk of both all-cause-related mortality and infection-related mortality [22]. Even in small children, the use of fistulae or grafts is associated with access survival rates equivalent to those of adults and with better survival rates than with cuffed venous catheters [23]. In a 20-year retrospective review of 304 vascular access procedures in children, the median survival time of arteriovenous fistulae was 3.1 years compared to 0.6 years for central venous access [24].

#### Peritoneal dialysis access and preservation of membrane function

It has become clear that a meticulous approach to PD catheter insertion by a dedicated team is, perhaps, more critical than the type of catheter or implantation technique used with, if possible, planned catheter placement before RRT becomes essential [20, 25]. However, the recent NAPRTCS data have shown that the time to the first episode of peritonitis is longer in children with two cuff catheters, swan-neck tunnels and for downward pointing exit sites [2]. Peritoneal membrane function is an independent predictor of patient survival, with those with high transporter status and, therefore, decreased ultrafiltration capacity demonstrating worse outcomes [26]. In a meta-analysis Brimble et al. showed an increased mortality risk of 21.9%, 45.7%, and 77.3% in low-average, high-average and high transporters, respectively, compared with patients with low transporter status [27].

As alterations in peritoneal membrane transport appear to be related to peritonitis episodes rather than the duration of dialysis, every effort must be directed to reducing peritonitis rates [28], including intensive training, flush-before-fill dialysis delivery systems, antibiotic prophylaxis for catheter insertion, and early treatment of exit site infections. Although there are no long-term data, the use of biocompatible PD solutions, i.e. normal pH, bicarbonate–lactate buffer, and low glucose concentrations, particularly in children who are anticipated to have a long wait on PD, may be advantageous. The use of icodextrin to increase fluid removal appears to be associated with less functional deterioration of the peritoneal membrane as the use of solutions with high glucose concentrations is avoided [29].

### Dialysis adequacy

#### Haemodialysis

Although the optimum haemodialysis dose has not been defined in children, the NKF K/DOQI and Renal Association guidelines state that children should receive at least the delivered dialysis dose as recommended for adults, i.e. either for a urea reduction ratio (URR) > 65% or an equilibrated Kt/V urea >1.2, delivered thrice weekly [19, 20]. The Hemodialysis (HEMO) Study trial in adults showed no difference in survival between patients with a mean eKt/V of 1.16 and those achieving a Kt/V of 1.53 [30]. These findings are similar to those in a recent study of 613 adolescents on HD, in which hospitalization risk was increased with a single pool Kt/V <1.2 compared to 1.2–1.4 but a spKt/V of >1.4 did not improve outcome [31]. However, in a smaller study of 12 children receiving a carefully controlled dietary intake and with a mean Kt/V of 2, URR 84.7% catch-up growth was demonstrated [32]. Increasing the frequency of HD sessions was shown to improve appetite significantly and to increase growth velocity in a recent small study of children and may lead to re-evaluation of dialysis adequacy in children [33].

#### Peritoneal dialysis

Current clinical opinion supports the recommendation that total solute clearance in paediatric patients should meet or exceed that in the guidelines for adults of a combined urinary and peritoneal Kt/V urea value per week of 1.8 [19] or 1.7 [10] or a creatinine clearance of 50 L/week per 1.73 m^2^ body surface area. In adult anuric patients a minimum peritoneal Kt/V urea value of 1.7 and an optimal target of 1.8 is suggested by Lo et al., based on survival data [34]. A previous interventional study by the same group clearly demonstrated increased clinical problems, including severe anaemia in patients with a total Kt/V of < 1.7, but no difference in survival outcome between patients with a Kt/V maintained above 2 and those with a value between 1.7 and 2.0, with the difference in Kt/V accounted for by increasing peritoneal clearance only [35]. The Adequacy of Peritoneal Dialysis in Mexico (ADEMEX) trial measured both peritoneal creatinine clearance and urea clearance as determinants of small-solute clearance and found no difference in 2-year survival rates between the control group and an intervention group with significantly greater clearances after adjusting for factors known to affect survival [36]. However, the Netherlands Cooperative Study on the Adequacy of Dialysis (NECOSAD) clearly identified an increase in the relative risk of death in anuric PD patients if the Kt/V urea value was < 1.5/week and creatinine clearance was < 40 L/week per 1.73 m^2^ [37]. There are no comparable mortality data for children, but the Network 1 Clinical Indicators Project reporting on clinical morbidity in paediatric dialysis patients found that, in a small group of well-dialysed patients on either HD or PD, the exceeding of recommended adequacy guidelines did not influence morbidity [38]. Indeed, in children on PD, a Kt/V value of > 2.75 was associated with increased albumin losses, which may have an adverse effect on nutrition and growth [39].

### Residual renal function

Although the above studies have attempted to describe an optimal dialysis dose with reference to dialysis adequacy, it is clear from adult studies that residual renal function contributes significantly to patient survival. The CANADA-USA (CANUSA) trial [40] was important in defining adequacy of small-solute clearance, but re-evaluation of the data identified that residual renal clearances was a more important factor for survival than was peritoneal clearance and combined ultrafiltration [41]. This has been confirmed in adult HD and PD patients in the NECOSAD studies [37], and it is recommended that the dialysis prescription take into account residual renal clearances. A smaller study in children suggests a correlation between renal solute clearance rather than peritoneal kt/V value and growth [42].

Studies of adults have shown that episodes of volume depletion, either unintentional or therapeutic, are associated with increased risk of loss of residual renal function [37, 43]. However, furosemide has been used successfully to achieve diuresis and improved fluid balance, without influencing residual renal function [44]. The use of angiotensin-converting enzyme inhibitors (ACEi) and angiotensin II receptor blockers in adult PD patients suggests a positive effect on preserving residual renal function [45, 46]. Results of the Effect of Strict Blood Pressure Control and ACE Inhibition on Progression of CRF in Pediatric Patients (ESCAPE) trial evaluating the effect of ramipril on estimated glomerular filtration rate (eGFR) and proteinuria in children with CKD, including a small cohort of PD patients, are awaited.

### Nutrition and growth

The prevention of malnutrition and associated hypoalbuminaemia is critical to the improvement of long-term outcome and the achievement of optimal growth in children on dialysis. In a recent teaching article for *Pediatric Nephrology*, Rees and Shaw discussed at length the importance of nutrition and growth in CKD patients [47]; however, some pertinent points regarding the long-term outcome are mentioned here.

Wong et al. found a significant association between hypoalbuminaemia and mortality in 1,723 patients aged < 18 years starting dialysis, with each −1 g/dl difference in serum albumin between patients associated with a 54% higher risk of death, after adjustment for glomerular causes and other known risk factors [13]. Poor nutritional intake occurs early in chronic renal failure (CRF), with deterioration in anthropometric indices as renal function deteriorates [48]. A low standard deviation score (SDS) for height at the start of dialysis is associated with an increased risk of death, as shown by Wong et al. and Furth et al., with a 14% increased risk of death with each decrease of 1 SDS for height [49], and a height SDS of < 2.5 associated with a doubled risk of death [50], respectively. Thus, early nutritional intervention may be important both for long-term survival and linear growth.

Although the NAPRTCS registry data show a decrease in height SDS, for those on dialysis, from −1.64 [standard error (SE, 0.03)], to −1.71 (0.04) at 1 year and −1.84 (0.05) at 2 years [2], we have demonstrated that an early and more intensive approach to feeding maintains or even improves height SDS [8, 14, 51]. In our experience, 89% of children that presented before they were 2 years old with CKD stages 4– 5, who subsequently underwent dialysis, required enteral feeding. Their mean height SDS improved from −2.18 (SD 1.44) at 6 months to −1.74 (1.55) at 1 year and −1.51 (1.38) at 2 years and improved steadily to −0.87 (1.51) at 5 years [51]. None of these children was treated with growth hormone. However, the use of recombinant human growth hormone (rhGH) remains controversial; some studies have shown that a combination of dialysis under adequacy control and careful attention to nutrition can promote normal growth [52], while others support the use of rhGH in selected cases [53].

### Psychosocial outcome

Young adults with childhood-onset CKD stage 5, particularly those who have had a longer period of time on dialysis, are more likely to have cognitive and learning impairment than is an age-matched population [54]. Bawden et al. performed neuropsychological assessments in sibling pairs and showed that, although the children with CKD 5 had mild deficits of IQ and fine motor coordination, encouragingly, there were no differences in measures of academic achievement, memory, behaviour or self-esteem [55]. More recent studies using health-related quality of life indices have shown that children with CKD have lower scores than healthy controls, but, surprisingly, children on dialysis have higher scores than would be expected, compared to transplant patients [56, 57]. In support of this, Groothoff et al. have shown that, although survivors of prolonged dialysis during childhood are twice as likely to be unemployed than an age-matched population, and taking into account the unavoidable physical problems, the overall subjective health perception of these young adults is surprisingly good [58]. Addressing the emotional, educational and social needs of children on dialysis by the provision of psychosocial and teaching support plays a crucial role in improving well-being and survival outcome, and must form an integral part of patient care.

### Cardiovascular risk factors

With improvements in renal replacement therapy, cardiovascular disease is increasingly recognized as a life-limiting problem in young patients with CKD, giving a 1,000-times higher risk of cardiovascular death than in the healthy age-adjusted population [59]. Analysing the USRDS database, Parekh et al. reported that 311 of the 1,380 (22.5%) deaths in patients aged 0–30 years who had undergone dialysis between 1990 and 1996 were from a cardiac cause [60]. Similarly, ANZDATA [3], the Dutch cohort [4], and German [11] and Polish [61] single-centre studies reported cardiovascular disease as the single most common cause of death in their CKD patients. Chavers et al. presented the largest study on cardiovascular morbidity in children on dialysis from the Medicare database [62]. In 1,454 incident paediatric dialysis patients aged 0–19 years, who underwent dialysis from 1991–1996, 452 (∼31%) developed a cardiac-related event. Arrhythmias (19.6%), valvular disease (11.7%), cardiomyopathy (9.6%) and death from cardiac arrest (3%) were reported. Unlike adults with CKD in whom coronary artery disease is the leading cause of death, cardiac arrest is the most commonly reported cause of death in children [60].

**Fig. 2:**
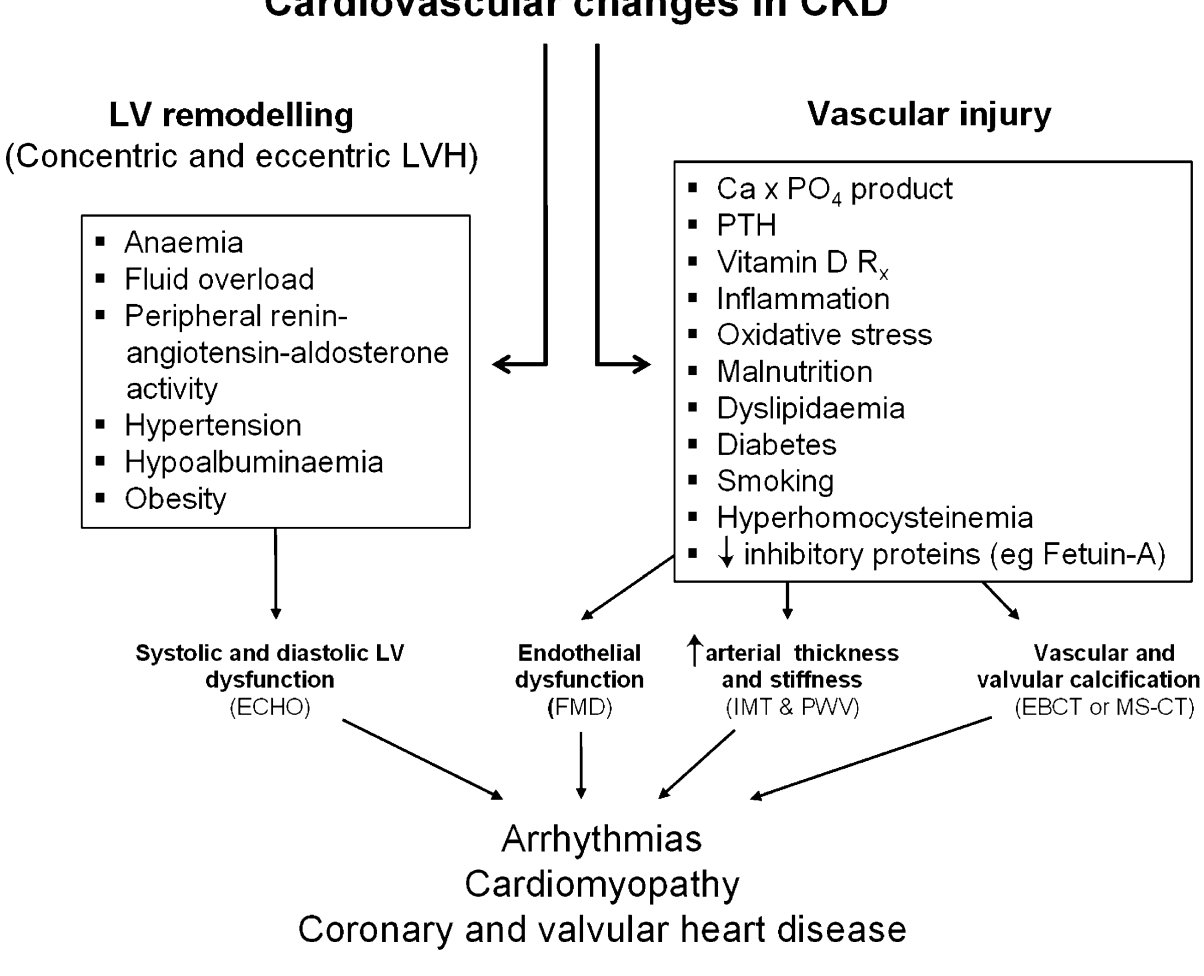
Cardiovascular changes, underlying risk factors, investigations for assessment and the clinical outcomes in patients with CKD, *ECHO* Echocardiogram, *FMD* Flow mediated dilatation, *IMT* Intima media thickness, *PWV* Pulse wave velocity, *EBCT* Electron Beam Computed Tomography, *MS-CT* Multi-Slice Computed Tomography

The ‘traditional’ risk factors, such as diabetes and dyslipidaemia, cannot account for the greatly increased risk of cardiovascular disease in children with CKD [63]. CKD presents a host of metabolic, mechanical and inflammatory damage-inducing agents, such as mineral imbalance [64, 65] associated with secondary hyperparathyroidism [66, 67], inflammatory mediators [68, 69], oxidative stress [70, 71], hyperhomocysteinaemia [72, 73], hypoalbuminaemia [74], dyslipidaemia [75], anaemia [76] and chronic fluid overload [77, 78]. These factors, acting individually or in concert, result in endothelial dysfunction [72, 73, 79], arterial stiffness [80, 81], and calcification [82], which contribute to cardiac remodelling with left ventricular hypertrophy (Fig. 2). In a recent educational feature, Mitsnefes comprehensively discussed cardiovascular morbidity in children with CKD [83], but some salient risk factors are discussed below.

#### Fluid overload and hypertension

Using the NAPRTCS database, Mitsnefes et al. have shown that 57% of children on long-term dialysis have a blood pressure (BP) above the age-, gender- and height-specific 95th centile [84]. In 51% of patients the BP was > 95th centile after 1 year of dialysis, and it had not changed significantly after the first year [84]. Also, the pulse pressure (= systolic−diastolic BP), which reflects the arterial wall compliance, has been shown to be a significant risk factor for cardiovascular mortality in adults [85].

Chronic volume overload is the most important factor contributing to uncontrolled hypertension in the dialysis population [60], with significantly higher BP in HD patients than in PD patients reported in all series [60, 78, 86–88]. A single ‘office’ BP may underestimate the true prevalence of hypertension, and ambulatory BP monitoring is increasingly used to diagnose and manage hypertension in dialysis patients [88–90].

#### Anaemia

The 2001 NAPRTCS annual report showed that 63% of children on long-term dialysis were anaemic after 6 months of dialysis, despite routine use of erythropoietin (EPO) [haemoglobin (Hb) levels < 11 g/dl] [91]. Warady and Ho report a 52% increased risk of death in association with the presence of anaemia, with cardiopulmonary or infectious diseases being the most common causes of death [92]. Recent data from the UK Renal Registry (9th report) support this observation: 47% of children on dialysis in the UK have an Hb < 11 g/dl [93]. Recent studies of adults, reporting increased morbidity associated with Hb levels > 12 g/dl [94, 95], have led to a recent revision of the K/DOQI guidelines that now recommend that Hb levels be maintained at > 11 g/dl and < 13 g/dl [96].

Long-standing exposure to the above risk factors leads to abnormal left ventricular (LV) remodelling, and left ventricular hypertrophy (LVH) has been reported in 30–80% of children on dialysis [77, 97–99], with a higher incidence in HD patients than in PD patients [98]. Two distinct patterns of LV remodelling are seen in CKD patients: concentric LVH, resulting from pressure overload, as seen with hypertension [83, 100], and eccentric LVH that is related to volume overload, sodium retention and anaemia [83]. LVH leads to a decreased coronary reserve and arrhythmias [101], which, in turn, are responsible for a disturbing 1,000-fold increase in cardiovascular mortality in CKD stage 5 patients [59].

#### Vascular calcification

Calcification of the arterial media or Monckeberg’s sclerosis develops early in the course of CKD [102]. Studies of children with stages 2–4 CKD and on dialysis have shown that increased intima–media thickness of the carotid artery [61, 67, 103] (a measure of structural changes in the vessel wall), increased pulse wave velocity [67, 104, 105] (a measure of stiffness or loss of compliance of the vessel) and presence of coronary and valvular calcification on CT scan [67, 82, 106] are present as early as the first decade of life. Secondary hyperparathyroidism, with the associated increase in calcium and phosphate levels [64], and renal bone disease [107], as well as its treatment with calcium-based phosphate binders [61, 108] and vitamin D [61, 67, 103], have been implicated as major cardiovascular risk factors.

Studies of adults have shown that ∼65% have evidence of calcification even before starting dialysis, using calcium-based phosphate binders, or beginning vitamin D therapy [109]. Vascular calcification is accelerated on dialysis [11, 61, 103, 104, 106, 108]: worsening hyperparathyroidism and renal bone disease [63, 107], the presence of ‘damage-inducing’ inflammatory mediators, oxidative stress and advanced glycation end-products [68, 69], coupled with a loss of the naturally occurring inhibitors of calcification (such as fetuin-A) [110], all play a role in accelerating vascular calcification. The time on dialysis is a strong independent predictor of vascular damage and calcification.

#### Dyslipidaemia

Abnormal lipid profiles are common in the childhood PD population but have not been extensively studied. Querfeld et al. reported hypertriglyceridaemia and hypercholesterolaemia in 69% and 90%, respectively, of children at the start of PD [75], with no significant change in the lipid profile during 2 years of dialysis. In a population of young PD patients, Scolnik and Balfe have shown that average serum cholesterol and triglyceride levels were 27% and 122% higher than the age-related normal levels [111]. Also, peritoneal losses of the low molecular weight lipoproteins results in lower levels of the protective high-density lipoproteins (HDLs) [75, 112]. In a similar study of adults the triglyceride, but not cholesterol, levels at initiation of dialysis were strong predictors of survival [113].

## Summary

In summary, although dialysis in children carries an increased mortality rate, meticulous care to reduce modifiable risk factors is important in this group, who have a lifetime of renal replacement ahead of them.

## Questions

(Answers appear after the reference list)

1. Which one of the following statements regarding residual renal function (RRF) is true:
  A. Once a patient is on dialysis there is no reason to try and preserve RRF
  B. Maintenance of RRF is of importance only for patients on peritoneal dialysis and not for those on haemodialysis
  C. Angiotensin-converting enzyme inhibitors (ACEi) have a significant benefit in preserving RRF compared to calcium channel blockers for an equivalent blood pressure control effect
  D. Use of icodextrin allows better preservation of RRF
  E. The use of diuretics in peritoneal dialysis patients is associated with an improvement in the RRF
2. Which one of the following statements regarding cardiovascular disease (CVD) in children with chronic kidney disease is false:
  A. An elevated level of C-reactive protein (CRP) is a risk factor for CVD
  B. Children with CKD develop CVD because of a higher prevalence of traditional risk factors such as diabetes and dyslipidaemia
  C. Low serum albumin levels influence cardiovascular outcome
  D. CVD is the single most common cause of death in young dialysis patients
  E. Patients with CKD can develop vascular calcification even without the use of calcium-based phosphate binders or Vitamin D treatment
3. An 18-year-old boy with posterior urethral valves has had two failed renal transplants and been on continuous cyclic peritoneal dialysis (CCPD) for ∼6 years. He has been anuric for the past 4 years. Over the past 6 months he has developed gradual-onset oedema and reduced ultrafiltration volume, although the catheter appears to work well. Which one of the following investigations or treatment options is not appropriate:
  A. Change to a bicarbonate-buffered dialysate in order to preserve the peritoneal membrane function
  B. Use icodextrin for a daytime dwell
  C. Start therapy with an angiotensin-converting enzyme inhibitor (ACEi) in order to preserve residual renal function
  D. Perform an abdominal CT scan with enteral contrast agent
  E. Perform a 3.86% dextrose dwell time and obtain a 4 h drain volume and measure serum and dialysate sodium, glucose and creatinine at 0 h, 1 h, 2 h and 4 h
4. For the patient described in the above question, which one of the following statements is most likely when this patient is aged 30 years:
  A. He has developed type 1 diabetes
  B. He has had a myocardial infarction
  C. He is still on peritoneal dialysis
  D. He is in full-time employment
  E. He has two children
5. A 4-week-old term baby with posterior urethral valves and bilateral dysplastic kidneys has a maximum creatinine concentration of 400 μmol/l (4.5 mg/dl) at 2 weeks of age and is started on peritoneal dialysis. Which of the following statements suggests a poor long-term prognosis:
  A. He has microcephaly
  B. His systolic blood pressure is 100 mmHg
  C. A postnatal renal ultrasound scan shows kidney sizes of 6 cm and 8 cm (50th centile for age and height = 5 cm), with multiple small cysts
  D. His mother has grade 3 vesicoureteric reflux
  E. He develops peritonitis within 1 week of commencing peritoneal dialysis
6. A 14-year-old boy presents to his local accident and emergency (A&E) unit with vomiting and lethargy and has a creatinine concentration of 500 μmol/l (5.7 mg/dl). Which one of the following interventions is likely to slow the progression to end-stage renal disease:
  A. Dietary protein restriction
  B. Use of a statin
  C. Maintenance of normal blood pressure
  D. Maintenance of Hb >13 g/dl
  E. Maintenance of a normal fluid allowance
